# A Novel Indicator of Plantar Fasciitis from Ultrasound Examination: A Cross-Sectional Cohort Study

**DOI:** 10.3390/jcm15197520

**Published:** 2026-09-27

**Authors:** Chun-Ming Fu, Yu-Lin Tsai, Tung-Liang Lin, Yu-Chun Lee, Yuan-Yang Cheng

**Affiliations:** 1 Department of Physical Medicine and Rehabilitation, Taichung Veterans General Hospital, Taichung 407219, Taiwan; 2 School of Medicine, National Yang Ming Chiao Tung University, Taipei 112304, Taiwan; 3 Intelligent Long Term Medical Care Research Center, Department of Post-Baccalaureate Medicine, College of Medicine, National Chung Hsing University, Taichung 402202, Taiwan

**Keywords:** plantar fasciitis, Achilles tendon, plantar fascia, diagnostic imaging, ultrasound, clinical assessment, morphometrics

## Abstract

**Background/Objectives:** Plantar fasciitis and Achilles tendinopathy are clinically linked conditions that share numerous biomechanical predispositions; however, the sonographic morphological correlations between the plantar fascia (PF) and Achilles tendon (AT) remain underutilized in routine diagnostic algorithms. The primary objective of this study was to investigate the concurrent relationship between the ultrasound morphometrics of the PF and AT in patients with plantar fasciitis. The secondary objective was an exploratory assessment to evaluate whether AT measurements can serve as an auxiliary diagnostic tool. **Methods:** A cross-sectional diagnostic study was conducted, evaluating 45 adults with clinically and ultrasonographically confirmed unilateral plantar fasciitis. The evaluation encompassed 45 affected feet and 45 contralateral unaffected control feet (internal controls). The main outcome measures included ultrasonographic PF thickness, AT thickness, and AT width. Statistical evaluations utilized the Wilcoxon signed-rank test for paired comparisons, Spearman correlations, Generalized Estimating Equations (GEE) multivariable logistic regression to account for paired-limb data clustering, and receiver operating characteristic (ROC) curves. **Results:** AT thickness and width correlated moderately, yet significantly, with PF thickness (r_s_ = 0.49 and 0.59, *p* < 0.001). ROC analysis demonstrated that an AT thickness >0.43 cm achieved 86.0% sensitivity, 48.1% specificity, and an area under the curve (AUC) of 0.71. Furthermore, an AT width >1.48 cm yielded 85.2% specificity and an 88.9% positive predictive value. GEE multivariable logistic regression adjusted for age and gender and accounting for within-subject clustering revealed that an AT thickness >0.43 cm was significantly associated with a 6.99-fold increase in the odds of having concurrent plantar fasciitis (Adjusted OR 6.99, 95% CI: 2.05–23.89, *p* = 0.002). **Conclusions:** Ultrasonic AT thickness and width exhibit a moderate, significant morphological association with PF thickness. An AT thickness >0.43 cm serves as a valuable supplementary diagnostic indicator associated with plantar fasciitis, underscoring the clinical importance of evaluating the entire enthesis complex concurrently.

## 1. Introduction

Plantar fasciitis is the most common cause of plantar heel pain in adults [1], fundamentally characterized by fascial thickening and degenerative tissue changes [2]. Plantar fasciitis is disproportionately common in individuals with structural foot deformities, such as leg length discrepancies, pes planus, and pes cavus, which are intricately linked to tightness of the Achilles tendon (AT) [1,3]. Recognized independent risk factors for plantar fasciitis encompass reduced ankle dorsiflexion, elevated body mass index, and occupational weight-bearing activities [3]. Concurrently, restricted ankle dorsiflexion serves as a primary mechanical catalyst for the development of Achilles tendinopathy [4]. This shared biomechanical etiology and kinetic chain dysfunction underscore a highly synergistic clinical relationship between the plantar fascia (PF) and the AT.

Extensive research has demonstrated an explicit functional correlation between the tension of the PF and the AT. In cadaveric foot models, vertical compression tests have elucidated a significant association between increased strain on the AT and elevated pressure within the PF [5,6]. Overstretching of the AT, frequently driven by forceful contractions of the gastrocnemius muscle, has been associated with subsequent PF overload. This suggests that increased tension in the AT acts as a mechanical exacerbator for plantar fasciitis [5]. Furthermore, shear-wave elasticity sonography has revealed a strong correlation between AT stiffness and pain severity in patients with plantar fasciitis [7]. These findings emphasize the clinical imperative of evaluating the condition of the AT when diagnosing and managing plantar fasciitis.

Beyond biomechanical tension, morphological and structural changes in both the PF and AT are closely linked. Magnetic resonance imaging (MRI) studies of patients with Achilles tendinopathy have demonstrated a notable concurrent increase in PF thickness [8]. Similarly, recent cadaveric investigations have revealed a robust correlation between AT thickness and PF thickness precisely at the calcaneal insertion [9]. These dimensional correlations diverge significantly when comparing healthy tendons to those afflicted by tendinopathy [10]. Achilles tendinopathy, in particular, is pathologically characterized by progressive tendon enlargement, predominantly in its middle and distal segments [11]. The clinical coexistence of these two conditions is remarkably high: approximately 33% of plantar fasciitis patients concomitantly suffer from Achilles tendinopathy, whereas an overwhelming 81.8% of patients with Achilles tendinopathy are ultimately diagnosed with concurrent plantar fasciitis [12]. Furthermore, up to 63.8% of patients presenting with bilateral Achilles tendinopathy are simultaneously affected by plantar fasciitis [13]. This high prevalence of comorbidity underscores the necessity of a holistic, combined diagnostic approach in clinical practice, highlighting the value of utilizing accessible imaging tools like ultrasound to concurrently evaluate the PF and AT.

The utilization of ultrasound for the assessment of PF thickness has been widely validated and is considered highly reliable [14,15]. A PF thickness exceeding 4.0 mm, or a measurement >50% greater than the contralateral asymptomatic fascia, constitutes the current consensus diagnostic criterion for plantar fasciitis [16,17]. Ultrasound also serves as a crucial supportive modality for the diagnosis of Achilles tendinopathy [18]. A comprehensive ultrasound-based criteria set for Achilles tendinopathy has recently been proposed, identifying a mid-portion anteroposterior thickness >6 mm (reactive stage) or ≥8 mm (degenerative stage), coupled with the loss of the normal fibrillar echogenic pattern and the detection of intra-tendinous neovascularization on power Doppler [19]. Given that ultrasound is a widely accepted, highly accurate tool for assessing the morphometrics of both structures, this study leverages ultrasonography to quantitatively investigate the relationship between PF and AT thicknesses.

Despite the foundational insights into the PF-AT relationship provided by prior MRI investigations, few studies have translated these findings into practical applications for everyday clinical diagnosis. MRI is hampered by limited accessibility, long acquisition times, and high costs. Conversely, high-resolution ultrasound is cost-effective, readily accessible at the point of care, and allows for dynamic assessment. We hypothesize that the morphometric correlations between the PF and AT evaluated via ultrasound in living patients will mirror the structural relationships identified in cadaveric studies [9]. Specifically, patients with a pathologically thickened PF are expected to exhibit a correspondingly thicker AT, a hallmark of early or established Achilles tendinopathy.

While a PF thickness ≥4 mm on B-mode ultrasound is the most widely cited principal morphologic sign of plantar fasciitis, its stand-alone diagnostic accuracy presents limitations. Notably, up to 25% of heels that strictly meet the clinical diagnostic criteria for plantar fasciitis display a fascia <4 mm [20]. Conversely, 20% to 35% of entirely asymptomatic adults demonstrate a fascial thickness >4 mm [21,22]. A recent meta-analysis indicated that the classic 4-mm threshold yields a pooled sensitivity of only 66–78% and a specificity of 60–79% [23]. This diagnostic variability—heavily influenced by factors such as age, gender, body mass index (BMI), lifestyle, and daily standing/walking duration—exposes a critical need for complementary sonographic indicators. Incorporating AT morphometrics may help capture the broader enthesis-complex degeneration now recognized as integral to the pathology of plantar fasciitis.

Several auxiliary ultrasound markers have been proposed to address this diagnostic gap, including (i) shear-wave elastography to assess tissue stiffness, revealing 25–40% lower Young’s modulus in symptomatic tissues [24]; (ii) intrafascial or intratendinous neovascularity detected via power Doppler, correlating with pain phenotypes [25]; and (iii) heel-fat-pad (HFP) thickness evaluations (<12 mm or high compressibility), which help differentiate HFP syndrome from PF [26]. The primary objective of this study was to establish a quantifiable morphological association between the sonographic measurements of the PF and the AT in a cohort of patients with unilateral plantar fasciitis, utilizing their contralateral asymptomatic feet as internal matched controls. The secondary objective was to perform an exploratory assessment of the diagnostic performance of highly accessible AT morphometrics—specifically thickness and width—aiming to define optimal diagnostic cut-off values that can serve as reliable supplementary indicators for plantar fasciitis in routine clinical settings.

## 2. Materials and Methods

### 2.1. Ethics Approval

Ethical approval for this study was obtained from the institutional review board of Taichung Veterans General Hospital (Taichung, Taiwan; Protocol No. CE22536B, 31 January 2023). Patient consent was waived by the Institutional Review Board of Taichung Veterans General Hospital due to the minimal risk involved in this observational ultrasound study. The research was conducted in strict compliance with the Declaration of Helsinki and all relevant local and international ethical regulations.

### 2.2. Data Collection

This study utilized a cross-sectional cohort design with internal matched controls. To ensure the complete, transparent, and reproducible reporting of our diagnostic accuracy study, patient recruitment and the flow of participants through the index tests and reference standards were rigorously documented following the Standards for Reporting of Diagnostic Accuracy Studies (STARD) 2015 guidelines. A detailed flow diagram, illustrating the exact number of individuals eligible, enrolled, tested, and analyzed at each stage of the study, is provided in Figure 1.

The study population comprised patients who underwent diagnostic musculoskeletal ultrasound examinations for suspected plantar fasciitis at our outpatient physical medicine and rehabilitation clinic between 1 February 2023 and 31 December 2023. Given the paired nature of the study design (evaluating affected versus unaffected limbs within the same individual), sample size estimation was performed utilizing G*Power 3.1.9.7 for a Wilcoxon signed-rank test (matched pairs). Assuming a moderate effect size (d_z_) of 0.5, a two-sided α = 0.05, and a desired statistical power (1−β) of 0.80, the power analysis indicated a minimum requirement of 35 independent participants. To ensure robust statistical power, a minimum of 45 participants (yielding 45 matched pairs of feet) were targeted and successfully enrolled.

Inclusion criteria were: (1) chronic unilateral heel pain lasting >3 months; (2) local tenderness of heel pad or PF on physical examination; (3) reported history of the typical morning pain of plantar fasciitis; and (4) thickness of PF >4.0 mm or >50% thicker compared with the contralateral side, as based on ultrasound images in the longitudinal plane, which are the ultrasonic criteria for the current diagnosis of plantar fasciitis. (Supplementary Figure S1) [16,17]. Exclusion criteria were the incidence of ankle trauma during the recent 3 months, ankle or foot pain due to diagnoses other than plantar fasciitis, bilateral plantar fasciitis symptoms, and having systemic diseases with symptoms involving the ankle.

Demographic, anthropometric, and lifestyle data were systematically recorded, including age, gender, height, body weight, calculated BMI, and self-reported daily standing and walking durations. Based on occupational and daily habits, participants’ lifestyles were categorized binarily as either “mainly standing” or “mainly sitting”.

### 2.3. Ultrasound Scan

All sonographic evaluations were performed by a single experienced physiatrist to eliminate inter-rater variability. Ultrasound scans were conducted using either an E-CUBE i7 system (Alpinion, Seoul, Republic of Korea) equipped with a 3–12 MHz linear array transducer, or a LOGIQ P8 system (GE Healthcare, Chicago, IL, USA) utilizing a 6–15 MHz linear transducer. To eliminate potential intra-subject measurement bias arising from hardware variations, a strict protocol was enforced: for any given participant, both the affected foot and the contralateral unaffected foot were evaluated consecutively during an identical clinical session using the exact same ultrasound device. Participants were assigned to either the E-CUBE or LOGIQ system based purely on clinic room availability on the day of examination.

For the assessment of PF thickness, patients were placed in a prone position with their feet hanging over the edge of the examination table, knees fully extended, and ankles maintained in a neutral 90° dorsiflexed position. The transducer was aligned longitudinally over the medial aspect of the calcaneus, tracing a line from the calcaneal tuberosity toward the second toe. The probe was dynamically translated along the fascia to accurately identify the thickest point at the distal calcaneal edge (the enthesis) [16], as illustrated in Supplementary Figure S2a. To minimize measurement error, three independent measurements were taken, and the arithmetic mean was recorded for analysis.

For the assessment of AT parameters, the identical patient positioning was maintained. Starting at the calcaneal insertion, the transducer was tracked proximally along the longitudinal axis of the tendon up to the musculotendinous junction. The specific site of the maximal anteroposterior diameter—typically located 2 to 6 cm proximal to the superior border of the calcaneus—was identified. Following identification of the maximal thickness point, the probe was rotated 90° to obtain a transverse (short-axis) view. Only the true tendon proper was measured; the superficial lamina of the crural fascia and the echogenic paratenon were carefully excluded to prevent overestimation. Electronic calipers were placed precisely perpendicular to the anterior and posterior echogenic margins of the tendon. Three consecutive measurements of both thickness (anteroposterior diameter) and width (medial–lateral diameter) were recorded to the nearest 0.1 mm. The mean values were utilized for subsequent statistical analysis. If the variance between any two sequential readings exceeded 0.3 mm, the entire measurement sequence for that parameter was repeated to ensure maximal reliability (Supplementary Figure S2b) [27].

### 2.4. Statistical Analyses

Because the sonographic data exhibited a non-normal distribution based on the Shapiro–Wilk test, non-parametric statistical methods were employed. The sonographic parameters (PF thickness, AT thickness, and AT width) were compared between the affected (symptomatic) feet and the contralateral unaffected (asymptomatic) internal control feet utilizing the Wilcoxon Signed-Rank test for paired continuous data. The correlations between PF thickness and the measured AT parameters were evaluated using Spearman’s rank correlation coefficient (r_s_).

To evaluate the associative strength of AT parameters for the clinical diagnosis of plantar fasciitis while properly accounting for the clustered nature of the paired-limb design, Generalized Estimating Equations (GEE) were utilized. Treating paired feet from the same individual as independent observations violates standard logistic regression assumptions; therefore, a GEE binomial logistic regression model with a logit link function and an exchangeable working correlation matrix was constructed. Robust standard errors (sandwich estimators) were applied to compute accurate unadjusted and multivariable adjusted odds ratios (ORs) with 95% confidence intervals (CIs), adjusting for potential confounding variables, specifically age and gender.

Furthermore, Receiver Operating Characteristic (ROC) curve analysis was conducted to assess the overall exploratory diagnostic performance of the AT measurements. The Area Under the Curve (AUC), optimal sensitivity, specificity, positive predictive value (PPV), and negative predictive value (NPV) were calculated to identify the optimal diagnostic cut-off thresholds for AT thickness and width. All statistical analyses were performed using SPSS version 20.0 (IBM Corp., Armonk, NY, USA). The threshold for statistical significance was set at *p* < 0.05.

## 3. Results

### 3.1. Ultrasound Comparison of Plantar Fascia and Achilles Tendon Parameters

A total of 45 adult volunteers (20 male, 25 female) met all eligibility criteria and were enrolled in this study. This provided a matched cohort of 45 affected feet and 45 contralateral unaffected control feet for internal comparison. The median age of the cohort was 53 years (range: 21 to 91 years). The mean height was 164.1 ± 7.0 cm (range, 155 to 177 cm); and the mean body weight was 68.9 ± 14.4 kg (range, 50 to 104 kg).

Analysis of the sonographic measurements revealed highly significant structural differences between the symptomatic and asymptomatic sides. For PF thickness, the median value for the unaffected sole was 0.32 cm (Interquartile Range [IQR]: 0.27–0.35), whereas the affected sole demonstrated a significantly higher median value of 0.42 cm (IQR: 0.40–0.46) (*p* < 0.001). Similar pathological hypertrophy was observed in the Achilles tendon. The median AT thickness was 0.46 cm (IQR: 0.37–0.51) in the unaffected limbs, compared to 0.50 cm (IQR: 0.45–0.56) in the affected limbs (*p* < 0.001). Furthermore, the median AT width measured 1.33 cm (IQR: 1.21–1.46) on the unaffected side, significantly enlarging to 1.53 cm (IQR: 1.41–1.65) on the affected side (*p* < 0.001). These results, derived exclusively from the non-parametric analyses (Wilcoxon Signed-Rank test), underscore the concurrent structural enlargement of both the PF and AT in limbs afflicted by plantar fasciitis (Table 1).

### 3.2. Correlation Between PF Thickness and AT Parameters

Spearman’s rank correlation analysis was applied to quantify the continuous relationship between the thickness of the inflamed plantar fascia and the structural dimensions of the Achilles tendon. A statistically significant, positive linear correlation was identified between PF thickness and all measured AT parameters (Table 2).

Specifically, the correlation between PF thickness and AT width was moderate, yielding a Spearman’s rho (r_s_) of 0.59 (*p* < 0.001). Concurrently, PF thickness demonstrated a significant positive moderate correlation with short-axis AT thickness, yielding an r_s_ of 0.49 (*p* < 0.001). Scatter plots visualizing these data points (provided in Supplementary Figure S3) clearly illustrate the positive linear trajectory between fascial thickening and tendinous hypertrophy.

### 3.3. Diagnostic Accuracy of AT Parameters for Plantar Fasciitis

To determine the clinical utility of utilizing AT dimensions as an auxiliary diagnostic marker for plantar fasciitis, ROC curve analysis was performed (Figure 2, Table 3).

The analysis revealed that AT thickness is a highly sensitive diagnostic indicator. The AUC for AT thickness was 0.71 (95% CI: 0.590–0.804, *p* = 0.001). The mathematically optimal diagnostic cut-off point was identified at >0.43 cm. At this threshold, AT thickness provided a sensitivity of 86.0%, a diagnostic accuracy of 72.7%, a PPV of 75.4%, and an NPV of 65.0%. AT width demonstrated even stronger overall discriminative ability, yielding an AUC of 0.81 (95% CI: 0.710–0.894, *p* < 0.0001). The optimal cut-off point for AT width was identified at >1.48 cm. While slightly less sensitive, this measurement offered excellent specificity (85.2%) and a high PPV (88.9%). These ROC findings strongly support the integration of AT morphometric assessment into the standard diagnostic ultrasound protocol for heel pain. To facilitate clinical translation, a summary of proposed supplementary markers based on these thresholds is presented in Table 4.

### 3.4. Risk Factor Analysis and Multivariable Logistic Regression

The associative strength of AT parameters, alongside traditional demographic and lifestyle risk factors, was evaluated using GEE binary logistic regression models to properly adjust for within-subject clustering (Table 5).

In the unadjusted GEE model, traditional demographic variables failed to show a statistically significant association with the presence of plantar fasciitis. Age did not significantly influence risk (OR 1.02, 95% CI: 0.97–1.06, *p* = 0.482). Gender was similarly non-predictive, with males showing an OR of 1.08 relative to females (95% CI: 0.40–2.85, *p* = 0.880). Furthermore, variables traditionally assumed to exacerbate mechanical load—including height, body weight, BMI, daily standing time, walking time, and categorical lifestyle factors—did not demonstrate significant statistical associations within this matched cohort.

In stark contrast, structural parameters of the AT were highly associated with pathology. Patients exhibiting an AT thickness >0.43 cm possessed 5.7-fold higher odds of concurrently presenting with plantar fasciitis in the unadjusted model (OR 5.85, 95% CI: 1.90–17.12, *p* = 0.002). Crucially, after adjusting for potential confounders (age and gender) in the multivariable GEE model, the associative strength of AT thickness intensified dramatically: an AT thickness >0.43 cm was associated with a 6.99-fold increase in the odds of having plantar fasciitis (adjusted OR 6.99, 95% CI: 2.05–23.89, *p* = 0.002).

Similarly, an AT width >1.48 cm served as a potent independent correlate, showing a strong association with plantar fasciitis diagnosis (unadjusted OR 11.45, 95% CI: 3.15–38.50, *p* < 0.001) (adjusted OR 12.92, 95% CI: 3.48–47.90, *p* < 0.001). These regression analyses isolate tendinous structural hypertrophy as a primary, independent sonographic marker for plantar fasciitis.

## 4. Discussion

This study represents the first structured investigation to establish a direct, quantifiable morphological correlation between the sonographic thickness and width of the Achilles tendon (AT) and the presence of plantar fasciitis (PF) utilizing a matched internal-control methodology. High-resolution ultrasound is widely recognized as a highly reliable, accessible, and cost-effective modality for the structural assessment of the enthesis organ complex [19,28]. Our findings decisively demonstrate that the AT is consistently and significantly thicker and wider in limbs concurrently afflicted by PF. Consequently, we propose that increased AT thickness serves as a robust, independent auxiliary diagnostic marker. Specifically, clinicians should maintain a high index of suspicion for the presence or impending development of plantar fasciitis when a patient presents with an AT thickness exceeding 0.43 cm (4.3 mm). This threshold is particularly valuable in diagnosing patients who present with classic clinical symptoms of heel pain but display equivocal PF thickness measurements (e.g., <4.0 mm) on standard B-mode imaging. The significant positive linear correlations observed between PF thickness and both AT thickness (r_s_ = 0.49) and AT width (r_s_ = 0.59) harmonize seamlessly with the established anatomical, biomechanical, and advanced imaging literature. Anatomically, the PF and AT do not function as isolated units; they are linked across the posterior–inferior aspect of the calcaneus by continuous, force-transmitting collagenous periosteal fibers [9,29]. This interconnected fascial continuity facilitates seamless mechanical force transfer, playing a critical role in supporting and stabilizing the medial longitudinal arch of the foot during the gait cycle [30,31].

Biomechanical finite element models have corroborated this relationship, demonstrating that load-deformation vectors within the PF are directly correlated with tensile forces generated by the AT [32]. Experimental data reveals that a twofold increase in AT tension proportionally doubles the tensile strain experienced by the PF [5], with AT forces accounting for up to 65% of the total mechanical stress exerted on the plantar fascia in symptomatic patients [33]. Clinically, interventions aimed at stretching the gastroc-soleus complex and the AT effectively reduce pathological stress on the PF [34]. Consequently, clinical AT tightness is a dominant phenotype, observed in up to 80% of severe plantar fasciitis cases [33,35]. The frequent radiographic observation of concurrent posterior and inferior calcaneal spurs in these patients further underscores the chronic, bilateral tension experienced at this shared enthesis [36]. Prior MRI investigations align with our sonographic data, demonstrating increased AT cross-sectional areas in cohorts with chronic PF [37,38]. Furthermore, longitudinal studies have shown that structurally thickened (yet asymptomatic) Achilles tendons frequently transition into clinically symptomatic tendinopathy within a 12-month window [39]. Therefore, our ultrasound findings advocate for a paradigm shift: the sonographic assessment of Achilles tendinopathy markers should become a routine, mandatory component of the diagnostic and prognostic evaluation of plantar fasciitis. Our ROC analyses underscore the high diagnostic utility of these parameters (all AUC values >0.70). An AT thickness cut-off of 4.3 mm maximizes diagnostic sensitivity (86.0%) and accuracy (72.7%), whereas an AT width cut-off of 14.8 mm optimizes diagnostic specificity (85.2%) and PPV (88.9%). While these metrics offer immediate clinical utility, future large-scale, multi-center prospective validation is warranted to fine-tune these thresholds across diverse populations, accounting for physiological variations related to extreme height, gender, and elite athletic activity [11,40].

Interestingly, our multivariable logistic regression analysis revealed that traditional epidemiological risk factors—such as body weight, BMI, and cumulative occupational weight-bearing (standing/walking time)—did not exhibit statistically significant associations with the presence of PF in our cohort. While obesity (typically defined as BMI > 30 kg/m^2^) is frequently cited as a classical risk factor [3], our specific study population did not include morbidly obese individuals, which likely blunted this effect. Furthermore, despite the intuitive clinical assumption that prolonged standing directly induces plantar tissue microtrauma [3], several rigorous epidemiological studies support our findings, demonstrating no definitive causal link between cumulative weight-bearing hours and the onset of PF [41,42]. This divergence strongly suggests that localized structural and biomechanical alterations in tendon morphology—specifically, the tensile and structural integrity of the AT—are far more potent sonographic correlates of PF pathology than generalized demographic or lifestyle metrics. As our data demonstrates, a structural alteration yielding an AT thickness >0.43 cm elevates the concurrent likelihood of PF by a staggering 6.99-fold.

A defining methodological strength of this study is the utilization of the contralateral, asymptomatic foot as an internal matched control. We assert that this matched-pair intra-subject design provides a vastly superior methodological framework compared to traditional case–control studies utilizing separate cohorts of healthy volunteers [17,43]. By comparing the pathological state directly to the asymptomatic state within the identical physiological environment, we effectively neutralize a multitude of systemic confounding variables, including precise age, genetics, systemic inflammatory markers, gender, and total systemic mechanical loading [44]. This rigorous approach isolates localized structural pathology, maximizing the internal validity of the sonographic comparative analysis. By employing Generalized Estimating Equations (GEE) to statistically process this clustered, paired-limb data, we ensured that the resultant odds ratios reflect true pathological associations rather than artificially inflated Type I errors driven by intra-subject correlation.

The frequent co-occurrence of PF and Achilles tendinopathy [12,13] is driven by shared mechanical predispositions, including pes planus, leg length discrepancies, and overarching kinetic chain dysfunctions [3,45]. The tightness of the AT directly amplifies the risk of PF [46]. Under ultrasound, both pathologies manifest as localized tissue thickening and hypoechogenicity, yet universally accepted diagnostic thresholds for the AT remain a topic of debate [19,47]. Although PF thickness does not always perfectly correlate with subjective pain scores [48], tissue strain evaluated via shear-wave elastography shows a tighter correlation with nociception in both the PF and AT [7,48]. However, elastography currently suffers from a lack of widespread protocol standardization and limited availability in primary care settings, restricting its everyday clinical utility [49,50,51]. Conversely, B-mode measurement of tissue thickness is universally available, highly standardized, and correlates strongly with diminished ultimate tensile strength and aberrant strain responses [52,53]. Thus, basic morphometric assessment of AT thickness remains the most pragmatic and reliable clinical tool for early detection and treatment tracking.

Current musculoskeletal imaging guidelines strictly recommend evaluating the Achilles tendon in the transverse (short-axis) plane to ensure the ultrasonic beam remains perfectly perpendicular to the fibrillar architecture, thereby minimizing anisotropic artifacts. Reliability analyses consistently demonstrate that standardized short-axis measurements yield the highest precision and reproducibility [54]. The present study adheres strictly to these principles, revealing that a short-axis AT thickness exceeding 4.3 mm independently confers a highly significant diagnostic odds ratio for PF. This robustly supports the contemporary biomechanical theory that PF and AT degeneration represent a unified, continuous enthesis-complex disorder. In daily practice, a rapid, 10-s transverse sonographic sweep of the distal AT can rapidly identify patients whose PF falls below the classic 4 mm diagnostic threshold yet possess genuine, mechanically driven PF. This finding should immediately prompt clinicians to initiate targeted, early-stage rehabilitation protocols focusing on gastroc-soleus complex flexibility, eccentric loading exercises, and the prescription of night splints.

We acknowledge certain inherent limitations in our study design. Primarily, as a cross-sectional study, this research establishes morphological associations but cannot dictate temporal sequencing or causality; therefore, AT thickening cannot be definitively termed a predictive risk factor for the future onset of PF. Secondly, while utilizing the contralateral asymptomatic limb as an internal control robustly eliminates systemic confounders, it introduces the potential caveat of subclinical degeneration; the “unaffected” limb may harbor underlying mechanical stress due to kinetic chain compensation, potentially causing our measurements to underestimate the true pathological divergence compared to an entirely healthy population. Thirdly, our sample size, while statistically adequately powered based on our G*Power matching-pair analysis, represents a relatively small cohort drawn from a single institution. Fourthly, two distinct ultrasound machines were utilized during the study period. While intra-subject machine variability was completely eliminated by scanning both feet of a single participant with the exact same device consecutively, slight inter-machine calibration variances across the broader cohort remain a minor possibility. Finally, achieving absolute blinding of the sonographer to the symptomatic status of the patient’s foot was logistically unfeasible due to the interactive, real-time nature of clinical ultrasound evaluations and unavoidable patient feedback during positioning. To vigorously mitigate potential measurement bias, we enforced a strict, pre-defined scanning protocol relying exclusively on fixed bony landmarks. Furthermore, transducer pressure and angle were rigorously standardized to prevent artificial tissue compression and anisotropy. Future large-scale, prospective longitudinal studies incorporating advanced modalities like power Doppler, standardized shear-wave elastography, and the establishment of stratified normative databases are highly recommended to expand upon these foundational findings and determine the true temporal sequence of enthesis-complex degeneration.

## 5. Conclusions

This study confirms a highly significant, moderate positive morphometric correlation between the thickness and width of the Achilles tendon and the thickness of the plantar fascia utilizing high-resolution diagnostic ultrasound. In clinical practice, when an Achilles tendon thickness exceeding 0.43 cm (4.3 mm) is observed, the concurrent presence of plantar fasciitis should be highly suspected. Integrating Achilles tendon morphometrics into routine sonographic evaluations provides a highly sensitive, complementary diagnostic criterion. This dual-assessment approach is particularly invaluable for symptomatic patients who lack the classic, pronounced fascial thickening (>4.0 mm), enabling earlier, more targeted biomechanical interventions. However, given the cross-sectional nature of this investigation, these sonographic thresholds reflect concurrent morphological associations rather than predictive causality.

## Figures and Tables

**Figure 1:**
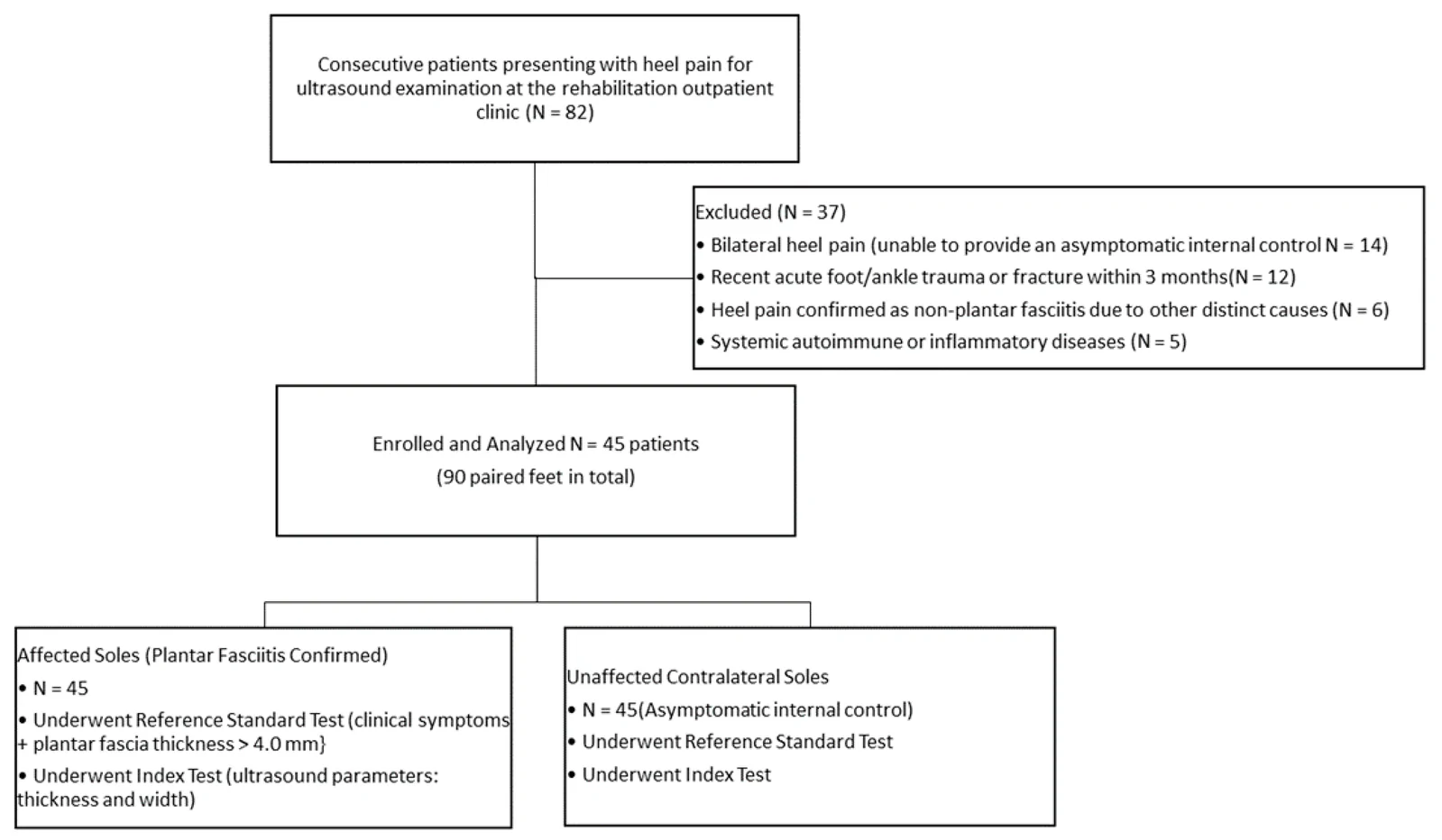
STARD 2015 flow diagram of participant recruitment. Of 82 patients assessed, 37 were excluded, leaving 45 patients (90 paired feet) for final analysis. Each patient served as an internal control comparing the affected sole to the contralateral asymptomatic sole.

**Figure 2:**
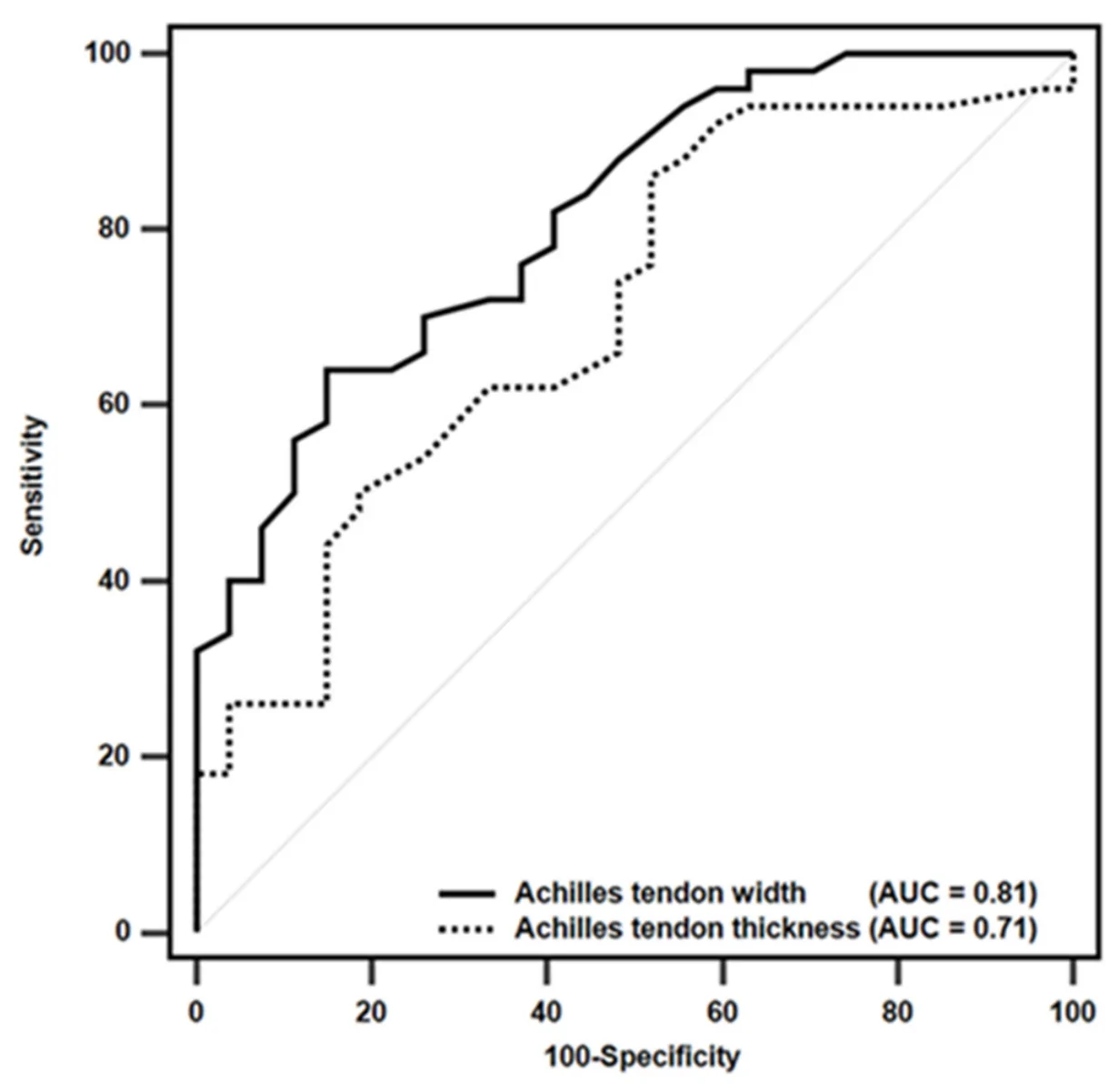
ROC curve analysis of Achilles tendon thickness and width in plantar fasciitis patients. The diagonal grey line represents the reference line of no discrimination (AUC = 0.5).

**Table 1:** Comparison of PF and AT parameters in plantar fasciitis patients.

	Unaffected Sole		Affected Sole		*p* Value
	Median	IQR	Median	IQR	
PF thickness (cm)	0.32	(0.27–0.35)	0.42	(0.4–0.46)	<0.001 **
AT thickness (trans)	0.46	(0.37–0.51)	0.50	(0.45–0.56)	<0.001 **
AT wideness (trans)	1.33	(1.21–1.46)	1.53	(1.41–1.65)	<0.001 **

Wilcoxon Signed Ranks test. ** *p* < 0.01. PF: plantar fascia, AT: Achilles tendon, IQR: interquartile range.

**Table 2:** Spearman’s correlation of PF thickness and AT parameters.

	PF Thickness (cm)		
	n	r_s_	*p* Value
AT thickness (long)	90	0.69	<0.001 **
AT thickness (trans)	90	0.49	<0.001 **
AT wideness (trans)	90	0.59	<0.001 **

** *p* < 0.01. r_s_: Spearman’s rho, PF: plantar fascia, AT: Achilles tendon.

**Table 3:** ROC curve analysis of AT thickness and width in plantar fasciitis patients.

Characteristics	AUC	95% CI	*p* Value	Cut-Off Point	Sensitivity	Specificity	Accuracy	PPV	NPV
AT thickness	0.71	0.590–0.804	0.00	>0.43	86.0%	48.1%	72.7%	75.4%	65.0%
AT width	0.81	0.710–0.894	<0.0001	>1.48	64.0%	85.2%	71.4%	88.9%	56.1%

ROC: receiver operating characteristic, AT: Achilles tendon, AUC: area under curve, CI: confidence interval, PPV: Positive predictive value, NPV: negative predictive value.

**Table 4:** OR of plantar fasciitis among literature-documented risk factors of plantar fasciitis and AT parameters.

Sonographic Marker	Diagnostic Utility	Clinical Interpretation
AT Thickness > 0.43 cm	High Sensitivity (86.0%)	Excellent tool for ruling out associated posterior compartment tightness when PF thickness is borderline (<4.0 mm).
AT Width > 1.48 cm	High Specificity (85.2%)	Strong confirmatory sign of enthesis-complex overload when heel pain symptoms are atypical.

**Table 5:** GEE Logistic Regression of Plantar Fasciitis Associations.

Variable	Unadjusted Model			Adjusted Model (Age and Sex)		
	OR	(95% CI)	*p* Value	OR	(95% CI)	*p* Value
Age	1.02	(0.97–1.06)	0.482			
Gender						
Female	1.00					
Male	1.08	(0.40–2.85)	0.880			
Height	0.96	(0.85–1.08)	0.850			
Body weight	1.02	(0.95–1.09)	0.650			
BMI	1.06	(0.92–1.22)	0.415			
Standing time/day	1.04	(0.70–1.55)	0.910			
Walking time/day	1.03	(0.68–1.50)	0.940			
Life style						
Mainly stand	1.00					
Mainly sit	1.05	(0.40–2.80)	0.920			
AT thickness > 0.43	5.85	(1.90–17.12)	0.002 **	6.99	(2.05–23.89)	0.002 **
AT width > 1.48	11.45	(3.15–38.50)	<0.001 **	12.92	(3.48–47.90)	<0.001 **

** *p* < 0.01. OR: odds ratio, CI: confidence interval, BMI: body mass index, AT: Achilles tendon.

## Data Availability

The raw data supporting the conclusions of this article will be made available by the authors upon reasonable request, subject to institutional privacy regulations.

## Supplementary Materials

The following supporting information can be downloaded at: https://www.mdpi.com/article/10.3390/jcm15197520/s1, Figure S1. Comparative sonograms of plantar fascia. Left panel: symptomatic foot with >50% thickening; right panel: asymptomatic contralateral foot showing normal thickness. Cal: calcaneus, Hollow arrowhead: plantar fascia. Figure S2. Measurement illustrations of plantar fascia (a) and Achilles tendon thickness/wideness (b) Cal: calcaneus, Hollow arrowhead: plantar fascia, Arrow: Achilles tendon. Figure S3. Scatter plots of plantar fascia thickness and three Achilles tendon parameters.
